# Synthesis, characterization and catalytic testing of MCM-22 derived catalysts for n-hexane cracking

**DOI:** 10.1038/s41598-020-78746-9

**Published:** 2020-12-11

**Authors:** Ali Ahmad, Salman Raza Naqvi, Muhammad Rafique, Habib Nasir, Ali Sarosh

**Affiliations:** 1 School of Chemical and Materials Engineering, National University of Sciences and Technology, H-12, Islamabad, 44000 Pakistan; 2 Department of Chemical Engineering, University of Gujrat, Hafiz Hayat Campus, Gujrat, Pakistan; 3 Quaid- I-Azam University, Islamabad, Pakistan; 4 Department of Chemistry, School of Natural Sciences, National University of Sciences and Technology, H-12, Islamabad, 44000 Pakistan; 5 Muhammad Nawaz Sharif University of Engineering and Technology (MNS-UET), Multan, Pakistan

**Keywords:** Chemistry, Materials science, Nanoscience and technology

## Abstract

Layered zeolites and their delaminated structures are novel materials that enhance the catalytic performance of catalysts by addressing diffusion limitations of the reactant molecules. n-Hexane catalytic cracking was observed over MCM-22 layered zeolite and its derivative structures over the temperature range of 450–650 °C for the production of olefins. MCM-22, H-MCM-22, and ITQ-2 zeolites were prepared by the hydrothermal method. Oxalic acid was used as a dealuminating reagent to obtain H-MCM-22 with various Si/Al ratios ranging from 09–65. The prepared samples were characterized by XRD, SEM, TGA, and BET. The cracking of n-hexane was carried out by Pyro/GC–MS. It was observed that the selectivity for olefins was improved by increasing the Si/Al ratio. H-MCM-22–10% produced the highest relative olefinic concentration of 68% as compared to other dealuminated structures. Moreover, the product distribution showed that higher reaction temperature is favorable to produce more olefins. Furthermore, a comparison between ITQ-2 and MCM-22 derived structures showed that ITQ-2 is more favorable for olefins production at high temperatures. The concentration of relative olefins was increased up to 80% over ITQ-2 at 650 °C.

## Introduction

Naphtha is an indeterminate product of several hydrocarbons between gasoline and benzene obtained as a byproduct from various petroleum crude sources with different physical properties instead of having a specific chemical composition containing approximately 6–10 carbon atoms per molecule. In naphtha, n-hexane is the principal constituent, and the typical composition of naphtha contains more than 30 wt% C_6_ compounds with the highest paraffinic composition of 53 wt%^1^. Naphtha is a product that contains heavy hydrocarbons that are less valuable. However, these heavy fractions can be converted to give more useful and valuable derivatives like olefins, which are the basic building blocks of plastic, textiles, paints, computers, and many other industries. Thermal cracking is the principal industrial method to produce olefins using naphtha and other organic feedstocks. However, thermal cracking is an energy-intensive process as it requires high temperature and steam operations.

Moreover, thermal cracking promotes CO_2_ emission, which is the primary cause of global warming. Besides, thermal cracking provides less control over product distribution, especially in the case of light olefins. Therefore, alternative technology considerably catalytic cracking is drawing much significance. The catalytic cracking of naphtha saves 20% more energy as compared to the conventional thermal steam cracking process and a reduction of nearly 20% CO_2_ emission^2^. Moreover, catalytic cracking of saturated hydrocarbons provides control over product distribution, especially for the production of light olefins.

In catalytic cracking processes, catalysts are used to enhance targeted product yield by providing more optimized operating conditions and improved energy transfer by providing continuous operation through regeneration and de-coking. For naphtha catalytic cracking, the most promising structures are zeolites with the three-dimensional porous structure of lattice cavities. These hydrated alumino-silicates constituted by eight, ten, or twelve membered ring oxygen atoms make small, medium, or large pores^3^. The zeolites help carry out catalytic cracking reactions at a lower temperature by lowering the activation energy as compared to thermal cracking. Zeolites, as compared to other catalysts, are highly resistive to thermal decomposition and can withstand the operating temperature of 150 °C less than steam cracking operating conditions^4^. Moreover, in the naphtha catalytic cracking process, zeolites can increase the olefinic selectivity up to 15% as compared to the conventional steam cracking process. Furthermore, zeolite lattice cavities range from 3 Å to 12 Å, which is the most suitable range for capturing naphtha crude saturated hydrocarbons for catalytic application residing within this limit.

In microporous zeolites, the selectivity of the catalyst is reduced due to limited catalytic efficiency as part of the volume ratio close to the outer surface takes part in the catalytic process engendering limited diffusion^5^. The diffusion resistance can be decreased by increasing pore diffusion and decreasing the diffusion path length. For this reason, microporous zeolites can be modified to obtain stable structures for olefins selectivity by dealumination using the hydrothermal treatment, ammonium hexafluorosilicate treatment, acid leaching and combination of steam acid leaching treatment^6–8^. Dealumination acts to remove extra framework aluminum (EFAL), causing as to increase lattice stability^9^.

In microporous zeolite structures, where the lattice cavities are less than 1 nm, the selectivity of zeolites towards cracking process is compromised. It allows less molecular diffusion of reactants in lattice structure; however, layered zeolites can be manipulated to enhance olefins selectivity. Mobil invented MCM-22 zeolite, a type of MWW zeolite with 10MR pore openings and layered structure with two independent pore channels^10^. One consists of two dimensional sinusoidal 10-MR slightly elliptical channels, and the other has a super cylindrical cage of 12-MR between layers. The outer surface crystals are formed by half super cages, which are accessible by 10-MR channels. MCM-22 gives rise to H-MCM-22 and poses many potential applications in cracking, alkylation, disproportionation, and isomerization reactions^11^. Meloni et al. devised the mechanism of n-heptane cracking over H-MCM-22 and suggested that cracking occurs in super-cages by classic carbenium ion chain mechanism. However, sinusoidal channels follow proteolysis^12^. The effect of catalytic cracking of n-hexane over dealuminated and delaminated (ITQ-2) counterpart of MCM-22 zeolite can be further investigated for olefinic production with the extent of dealumination by using acid treatment technique over the temperature range of 450 °C to 650 °C which is necessary to obtain high yields of olefins as a few papers are dealing with catalyst working at high temperatures above 600 °C.

MCM-22 zeolite can be synthesized with a very narrow range of Si/Al of about 10–30 for pure MCM-22 zeolite^13^. Several studies are available to synthesize MCM-22 zeolite via direct and post-synthesis techniques^13^. The post-synthesis technique provides a wide range of Si/Al of about 12–500 through structural conversion. Liu et al. prepared MCM-22 zeolites with different Si/Al ratios using the post-synthesis method and found that amount and strength of Bronsted acid sites are decreased with an increase in Si/Al ratio^14^. Another technique to increase the Si/Al ratio is dealumination. Wang et al. showed that the yield of light olefins could be increased by using higher Si/Al ratios of H-MCM-22 in n-hexane cracking^15^. The catalytic life and light olefins selectivity of the catalyst was improved using ammonium hexafluorosilicate (AHFS) as a dealuminating reagent. AHFS treatment restrains the bimolecular reaction of hydride transfer by decreasing Lewis acid sites, which in turn produces less coke. However, AHFS is not environmentally friendly, and alternative dealumination chemical or method such as hydrothermal treatment, acid treatment, the combination of steaming and acid leaching is required^6,7,16^. Matias et al. performed the dealumination of H-MCM-22 zeolite by nitric acid treatment and extracted 20% aluminum from the zeolite. The outer cups acid sites were deactivated entirely; however, the super cage structure was damaged. Wu et al. performed the dealumination of MCM-22 using a combination of steam action and acid treatment. The dealumination was relatively easy and caused selective removal of Bronsted acid sites on the external surface and 10-MR channels. However, the dealumination showed resistance to acid treatment due to highly stable MWW framework Al atoms even with 1 M HNO_3_ under reflux conditions. Wang et al. studied the catalytic properties and deactivation behavior of H-MCM-22 using oxalic acid as a dealuminating reagent^17^. Wang et al. performed the dealumination of the MCM-22 precursor before calcination using HNO_3_ and found that the tetrahedral aluminum atom on the T2 site in the hexagonal model was removed efficiently^18^. The dealuminated MCM-22 (Si/Al = 34) provided propylene selectivity of ca. 41%.

Besides the pore size increment techniques, layered zeolites can be delaminated by separation of layers to obtain high external surface area and enhanced catalytic activity for cracking applications. Moreover, large molecules in naphtha can access more active sites which are not easy to access in case of microporous catalyst where structural and size constraints are present. The layers separation provides the hexagonal array of cups created by the individual layers of 12 membered rings^19^. The joining of doubled six-membered rings provides a ten membered ring pore system at the center that runs throughout the structure in between the cups^20^. The catalytic cracking of n-decane and gasoline over ITQ-2 was studied by Corma et al.^21^. For both feeds, ITQ-2 provides higher olefins selectivity as compared to parent MCM-22 catalyst. Aguilar et al. also compared ITQ-2 with parent MCM-22 for alkylation of benzene and isomerization of m-xylene and found that ITQ-2 provides better activity for alkyl aromatics adsorption due to more accessible active site.

In this study, dealuminated and delaminated MCM-22 zeolites are prepared from MCM-22 precursor. The catalytic properties for n-hexane cracking are studied over hydrothermally synthesized MCM-22, oxalic acid treated H-MCM-22, and ITQ-2 as a model reaction for naphtha cracking to produce light olefins. Further, the extend of dealumination is investigated using different concentrations of oxalic acid. Finally, the comparison of n-hexane cracking over the prepared catalyst is discussed.

## Materials and methods

### Catalyst preparation

**Figure 1:**
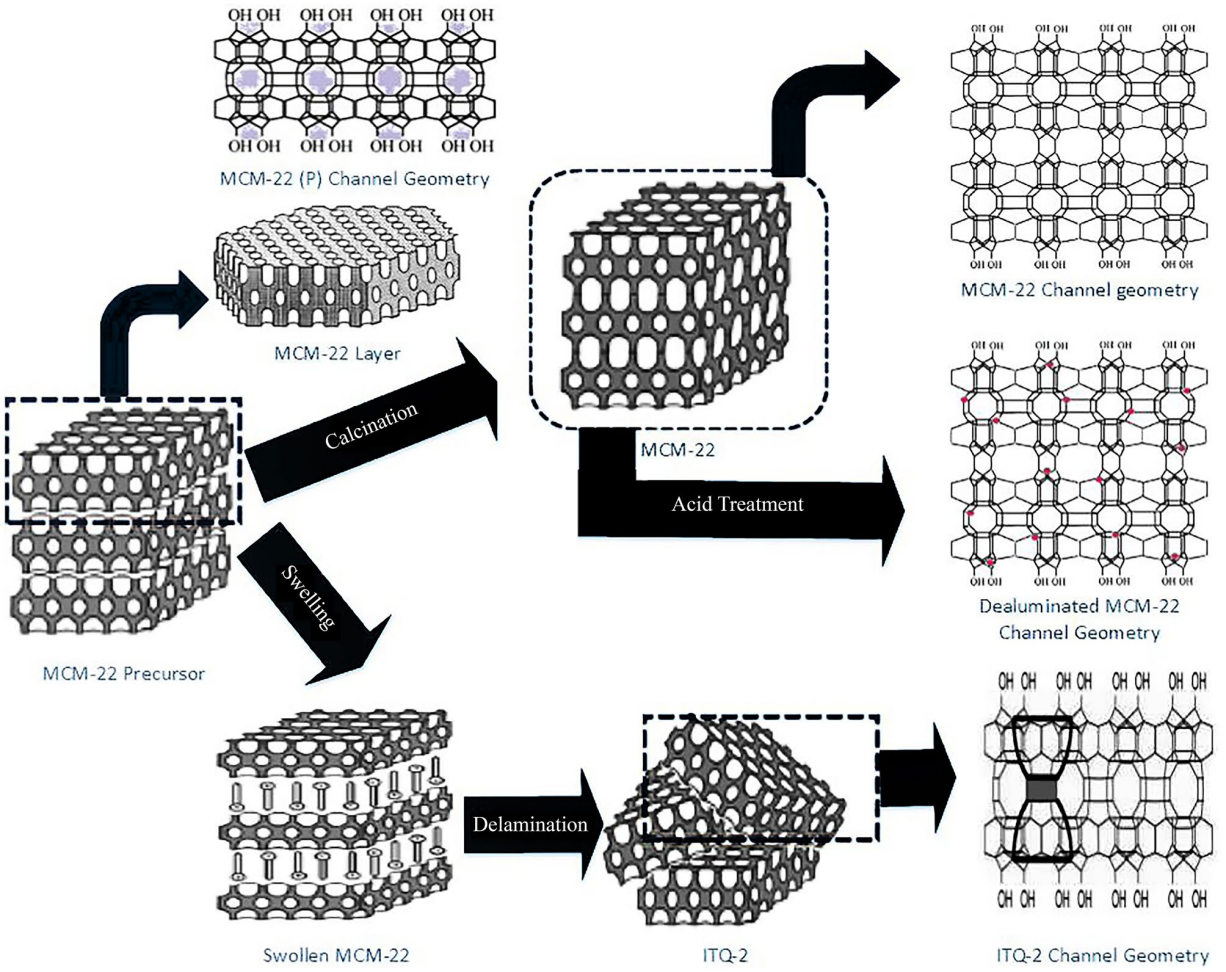
Scheme for the preparation of dealuminated and delaminated derivatives obtained from MCM-22 precursor^17,22^.

MCM-22 and ITQ-2 catalysts were prepared using the hydrothermal technique, as reported in the literature^21^. Figure 1 shows the scheme for the preparation of dealuminated and delaminated derivatives obtained from MCM-22 precursor. The synthesis mixture was prepared using sodium aluminate (56% Al_2_O_3_, 37% Na_2_O, Aldrich) sodium hydroxide (97%, Merck), silica (Aerosil 200, Degussa) and hexamethylenimine (99%, Aldrich). The solution of sodium aluminate and sodium hydroxide was made in deionized water. The resultant gel formed in this process was further treated for crystallization. The product was washed and dried to get MCM-22 precursor. After the calcination of the product, MCM-22 was obtained. MCM-22 was further ion exchanged with ammonium chloride (Merck 99.5%) and washed with HCl (1 M, Merck) to maintain its pH equal to 4.0. The obtained sample was calcinated and named as H-MCM-22. Further, the dealumination of H-MCM-22 was carried out by treating H-MCM-22 with different concentrations of oxalic acid (Sigma Aldrich) of 5%, 10%, 15%, and 20% solutions. Again, the process of calcination was performed to get the desired product, and the dealuminated samples obtained against 5%, 10%, 15% and 20% solutions of oxalic acid were designated as H-MCM-22 5%, H-MCM-22 10%, H-MCM-22 15%, and H-MCM-22 20% respectively. The final product ITQ-2 was finally obtained by swelling of MCM-22. The swelling was done by adding specific amounts of hexadecyltrimethylammonium bromide (98%, Sigma Aldrich) and tetra-propyl-ammonium-hydroxide (1 M, Sigma Aldrich) under proper reflux conditions. After the series of reactions, including sonication, washing with HCl to maintain pH = 2, drying, and finally, the calcination provided the final product denoted as ITQ-2.

### Characterization of the catalysts

Various characterization techniques characterized catalysts. The XRD patterns were recorded on a PANalytical Xpert pro (Germany model) XRD instrument using a Cu Kα X-ray source for shape and phase investigation of crystalline material. The N_2_ adsorption was carried out at 77 K on K-1042 (Costech International Italy) instrument to measure specific surface area, external surface area, and porosity. Thermogravimetric analysis (TGA) was carried out on a Q50 TGA (USA) analyzer in N_2_ atmosphere to measure the catalytic stability of prepared zeolites. The crystal size and morphology were examined with a JSM 6490 LV (JEOL) field-emission scanning electron microscope (FE-SEM).

### Catalytic cracking

The catalytic cracking of n-hexane on MCM-22 derived catalysts was carried out as a model reaction of n-hexane cracking. After the treatment of samples, the cracking with n-hexane in GC using a mass spectrometer detector (MSD) was performed to determine the pattern of the fragments. The catalytic cracking and product distribution results were carried out by using a pyro/GC–MS (Agilent Technologies, USA) under atmospheric pressure. Typically, the known quantity of catalyst was fed into the reactor, and the reactor temperature was adjusted as the desired temperature to activate the catalyst with airflow. 1 µl of n-hexane (AR grade) was diluted in helium and fed to the reactor with a flow rate of 0.1 ml/min. The capillary column separated all of the components of cracked n-hexane (DB-1 capillary column: ∅in = 0.25 mm, Length = 30 m) and detected through mass spectrometer detector (MSD). The percentage area of each component was shown on a chromatogram as the chromatographic peak. Based on the carbon number, the selectivity of the obtained products was calculated.

## Results and discussion

### Physiochemical characteristics of zeolites

#### XRD analysis

**Figure 2:**
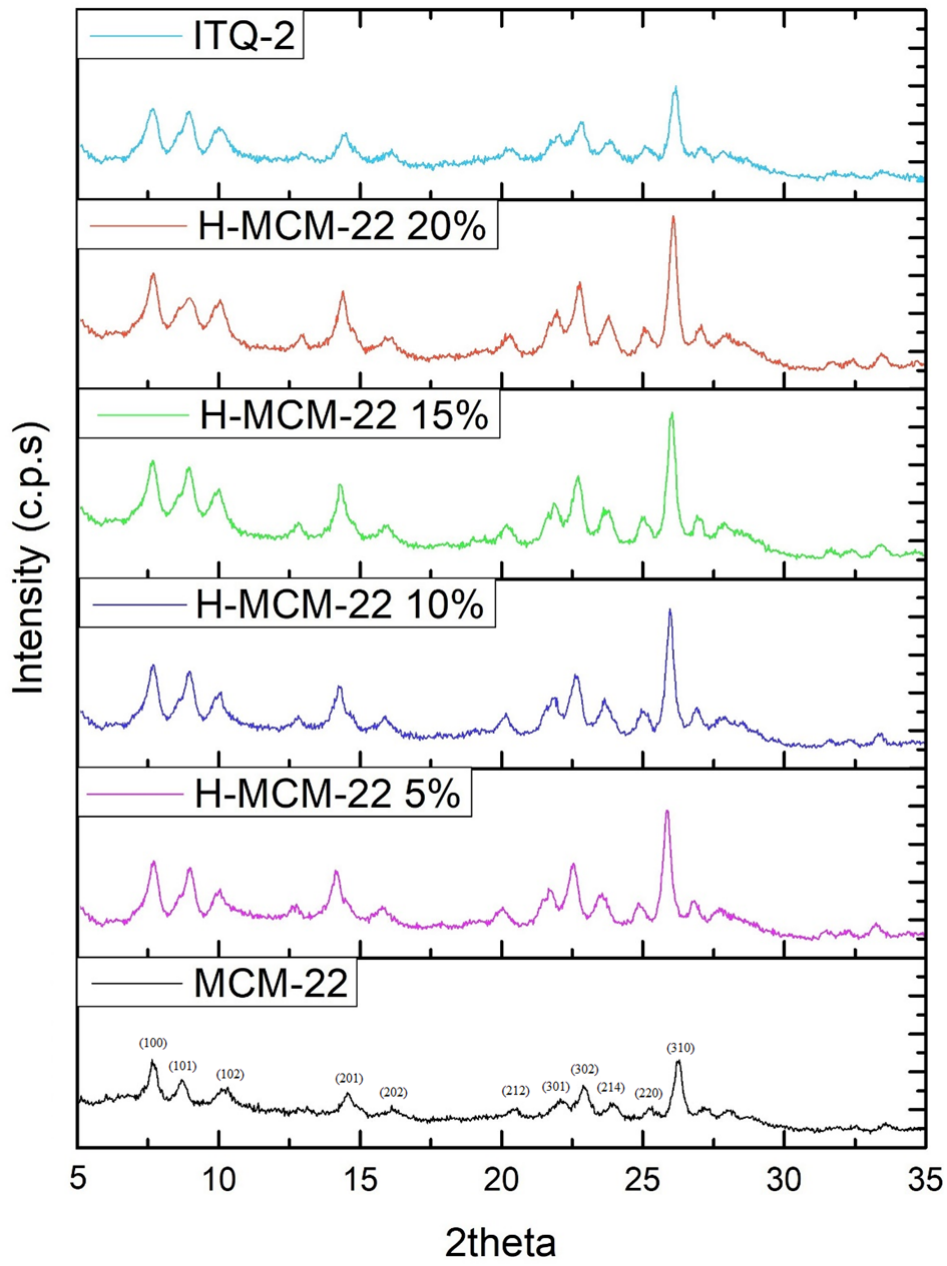
XRD patterns of ITQ-2, MCM-22, H-MCM-22 5%, H-MCM-22 10%, H-MCM-22 15%, and H-MCM-22 20% zeolites.

The synthesized samples in this cogitation has the MWW topographic composition and a relatively high crystallinity and contained no impurity of distinct phases as identified by XRD patterns. Figure 2 shows the XRD patterns for all prepared samples in a 2-theta range of 5°-35°. In precursor state (not shown here), vertically aligned layers are present, and 001 reflections represent layer separation while 002 reflection reflects d-spacing^23^. The calcination results in three-dimensional zeolite structure by the removal of template molecules and condensation of opposite external silanol groups. The black line in Fig. 2 is showing the XRD diffraction of MCM-22 with overlapping of interlayer reflection 002 with intralayer reflection 100. H-MCM-22 5%, H-MCM-22 10%, H-MCM-22 15%, and H-MCM-22 20% are represented by purple, blue, green, and red lines, respectively. As the concentration of oxalic acid is increased, the peak intensities of diffractograms increase without a change in a position providing an increase in lattice crystallinity by removal of EFAL.

The sky-blue line shows the Diffractogram for ITQ-2 in Fig. 2. The disappearance of reflection 002 with the convergence of 101 and 102 confirms the swelling while the disappearance of reflection 002 confirms delamination. Though diffractogram confirms the disordered layer structure, however, the internal zeolite structure is retained, which is confirmed by unchanged reflection 100. Moreover, the separation between the 101 and the 102 is explicit even after delamination providing 3D structured MCM-22 domains.

#### BET analysis

As shown in Table 1, the prepared H-MCM-22 and its dealuminated samples had moderately high BET surface regions in the specific range of 423–578 m^2^g^−1^, as determined with the aid of N_2_ adsorption, which revealed that they were of better quality and high crystallinity^13,17^. It can be seen that oxalic acid is an important and efficient dealumination reagent for H-MCM-22, and the Si/Al ratio is increased with an increase in the oxalic acid concentration from 5 to 20%. However, the crystalline structure of dealuminated zeolites is not affected by oxalic acid treatment, as confirmed by Fig. 2 and Table 1. In the case of dealuminated zeolites, as the concentration of oxalic acid is increased, the BET surface area and micropore volume decrease, which is consistent with the literature^24^. However, the external surface area is increased, which could be correlated with an increase in mesoporosity^24^. The nitrogen adsorption for the ITQ-2 sample shows a high external surface area of 700 m^2^/g, providing high mesoporous volume.

**Table 1:** BET surface area analysis of H-MCM-22, H-MCM-22 5%, H-MCM-22 10%, H-MCM-22 15%, and H-MCM-22 20%.

Catalyst	Si/Al	C_oxalic acid_	S_BET_ (m^2^/g)	V_micro_ (cm^3^g^-1^)
H-MCM-22	9	–	578	0.59
H-MCM-22 5%	14	5%	566	0.55
H-MCM-22 10%	22	10%	564	0.51
H-MCM-22 15%	41	15%	500	0.47
H-MCM-22 20%	65	20%	423	0.44

#### SEM

**Figure 3:**
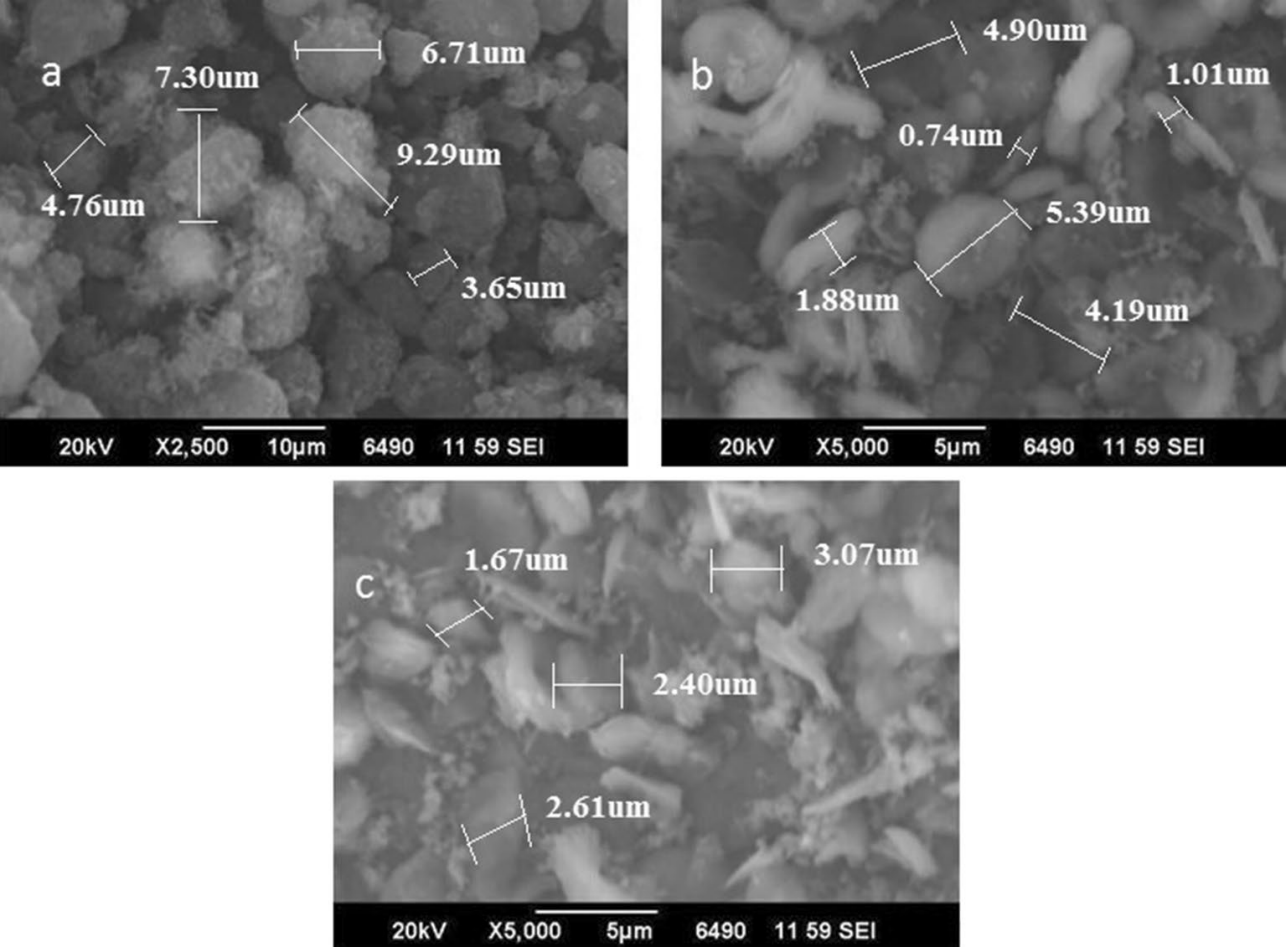
SEM image of MCM-22 (**a**), H-MCM-22 20% (**b**) and ITQ-2 (**c**).

The images regarding scanning electron micrographs (SEM) showed that samples H-MCM-22 and its derived samples were primarily composed of platelet like crystals, as shown in Fig. 3. The crystal size and morphology were not dependent on the ratio of Si/Al within the gels and synthesis technique and were unaffected by dealumination by acid treatment. MCM-22 morphology is beneficial as compared to other cracking catalysts in mass transfer operation^13^. ITQ-2 shows similar platelet morphology with a more aligned structure due to a single layer structure^25^.

#### TGA analysis

**Figure 4:**
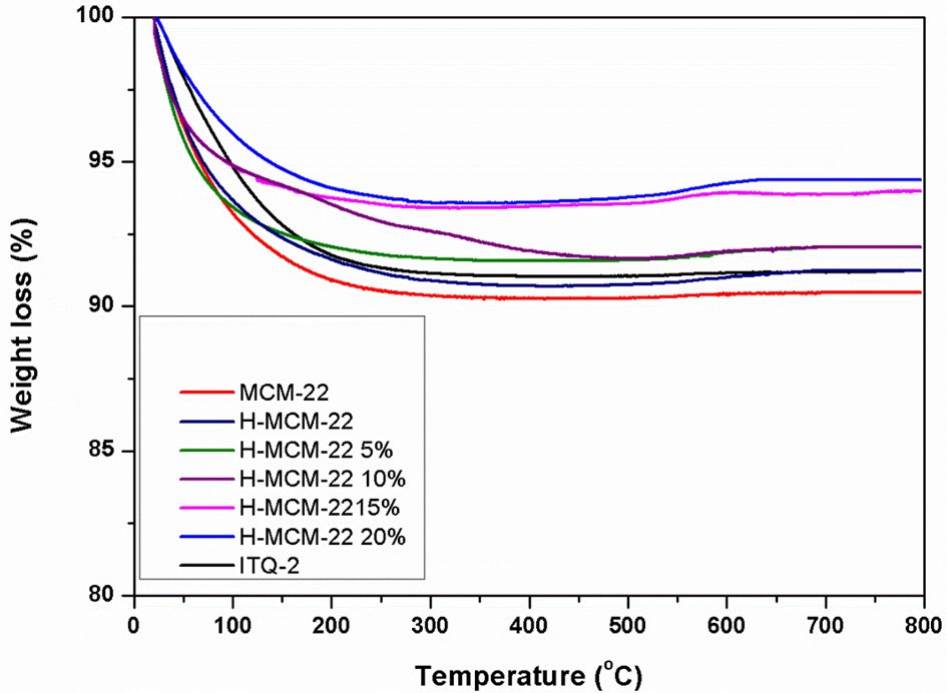
TGA analysis of MCM-22, H-MCM-22, H-MCM-22 5%, H-MCM-22 10%, H-MCM-22 15%, H-MCM-22 20%, and ITQ-2 zeolites.

TGA determined the percentage weight loss of spent catalysts for stability account. The TGA analysis of prepared samples showed that all the prepared catalysts are stable up to 800 °C as shown in Fig. 4. However, the stability for MCM-22 was further improved by dealumination, and the percentage weight loss was decreased for MCM-22 from 8.92% to 1.4% by dealumination. It is well established that the high amount of strong acid sites accelerates hydride transfer, aromatization, and other secondary reactions and become the major reason for coke formation and deactivation of the catalyst^26^. Dealumination removes acid sites, which suppresses the hydride transfer and coke formation^27^.

### Catalytic cracking

The products obtained from catalytic cracking of n-hexane were classified in two major classes of aliphatic and aromatic compounds while the relative olefins from aliphatic compounds were calculated using the formula:

$$Relative~Olefins = Olefins/(Olefins + Paraffins)$$

The selectivity of the products was determined based on the obtained carbon numbers.

#### Effect of reaction temperature

**Figure 5:**
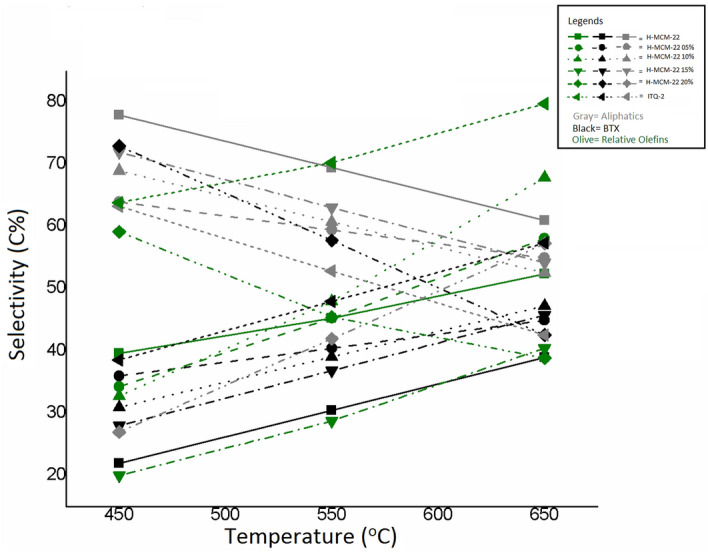
Effect of reaction temperature on the selectivity of products for catalytic cracking of n-hexane over prepared catalysts at different temperatures of 450 °C, 550 °C, and 650 °C.

The effect of reaction temperature over acid-catalyzed MCM-22 for catalytic cracking of n-hexane is shown in Fig. 5. The effect of temperature is studied over the temperatures range between 450 °C and 650 °C as the conversion of n-hexane is increased with an increase in temperature with a maximum conversion of 100% at 650°C^13^. The percentage conversion into aliphatic compounds was 78 percent at 450 °C that was reduced to 41% with an increase in temperature to 650 °C. The conversion of n-hexane into aliphatic compounds decreases with an increase in reaction temperature due to BTX (Benzene, Toluene, and Xylene) formation. However, the behavior of different hydrocarbons varies differently, with an increase in reaction temperature. For instance, in the case of olefins, propylene shows constant selectivity over the temperature ranging between 450 °C to 650 °C. Butenes and higher olefinic hydrocarbons show a decrease in selectivity due to subsequent conversion in BTX compounds. Ethylene following primary carbenium ion formation shows an increase in selectivity with an increase in reaction temperature^28^. Light alkanes, methane, and ethane follow monomolecular mechanism and their selectivity increase with the increase in temperature, however, in case of propane and butane, the selectivity decreases with an increase in reaction temperature as subsequent reactions engendering in BTX formation^29^. The product distribution and predominant monomolecular mechanism suggest a higher reaction temperature for n-hexane conversion and olefins production.

#### Effect of dealumination by oxalic acid treatment

**Figure 6:**
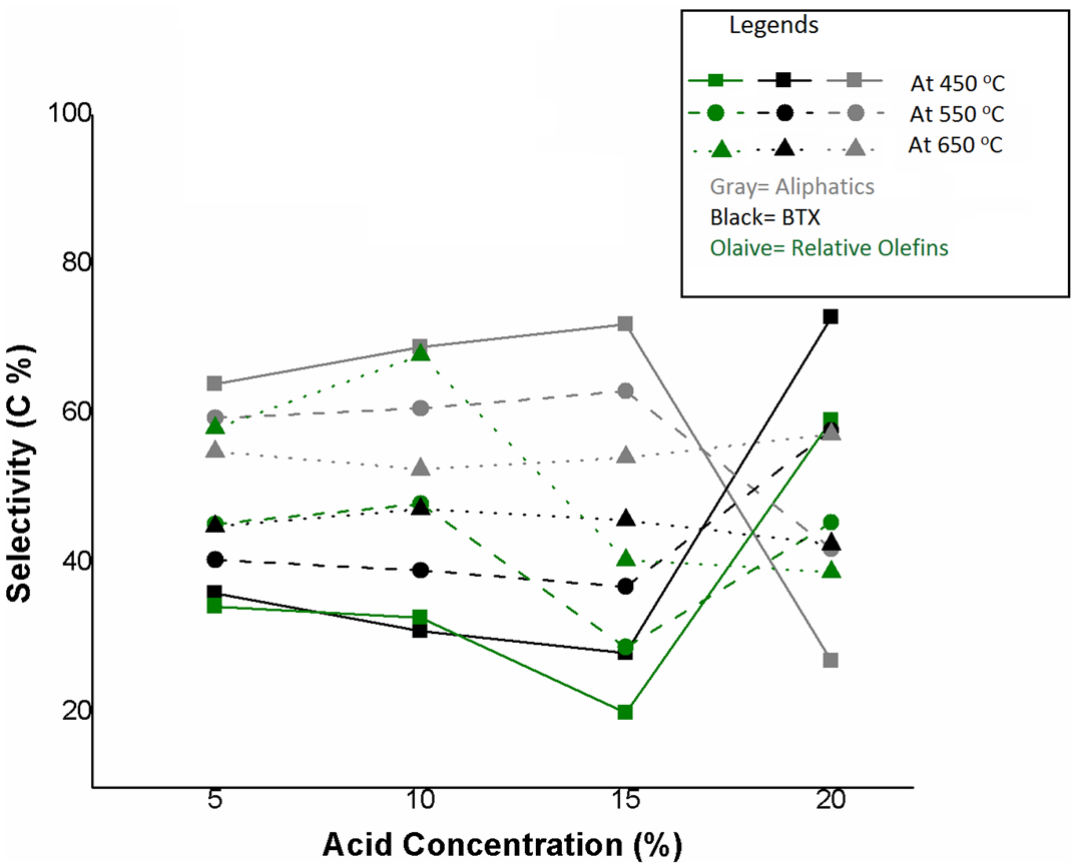
Effect of acid concentration on the selectivity of products for catalytic cracking of n-hexane over H-MCM-22 at different reaction temperatures of 450 °C, 550 °C, and 650 °C.

Oxalic acid is used as a dealuminating reagent, and its effect and extends on dealumination were investigated. The dealumination acts as to increase the Si/Al ratio by reducing Al content and hence reducing acid amounts^8^. Figure 6 shows the effect of dealumination on product distribution against different concentrations of oxalic acid. As the concentration of oxalic acid is increased, the Si/Al ratio increases, the olefinic selectivity increases up to 10% oxalic acid concentration while further increase in concentration tends to decrease olefinic selectivity, which determines the extent of oxalic acid concentration for dealumination of MCM-22. The higher concentration of oxalic acid engendered in the binding of surface hydroxyl groups. This binding causes the agglomeration followed by the fragmentation of crystal morphology^30^.

Further increase in acid concentration, beyond the dealumination extent, removed the active acid sites, and the process is predominantly facilitated by thermal cracking. Furthermore, BTX selectivity is slightly decreased, which suggests that BTX formation by cyclic aromatization, hydride transfer, and secondary reactions is suppressed due to a decrease in acid amounts^13^. In addition to this, a decrease in olefinic selectivity far off dealumination extent is also facilitated by the decrease in the acid amount, which reduced the subsequent cracking reactions of paraffin to produce olefins and resulted in increased paraffinic selectivity.

#### Catalytic cracking of n-hexane over dealuminated zeolites

**Figure 7:**
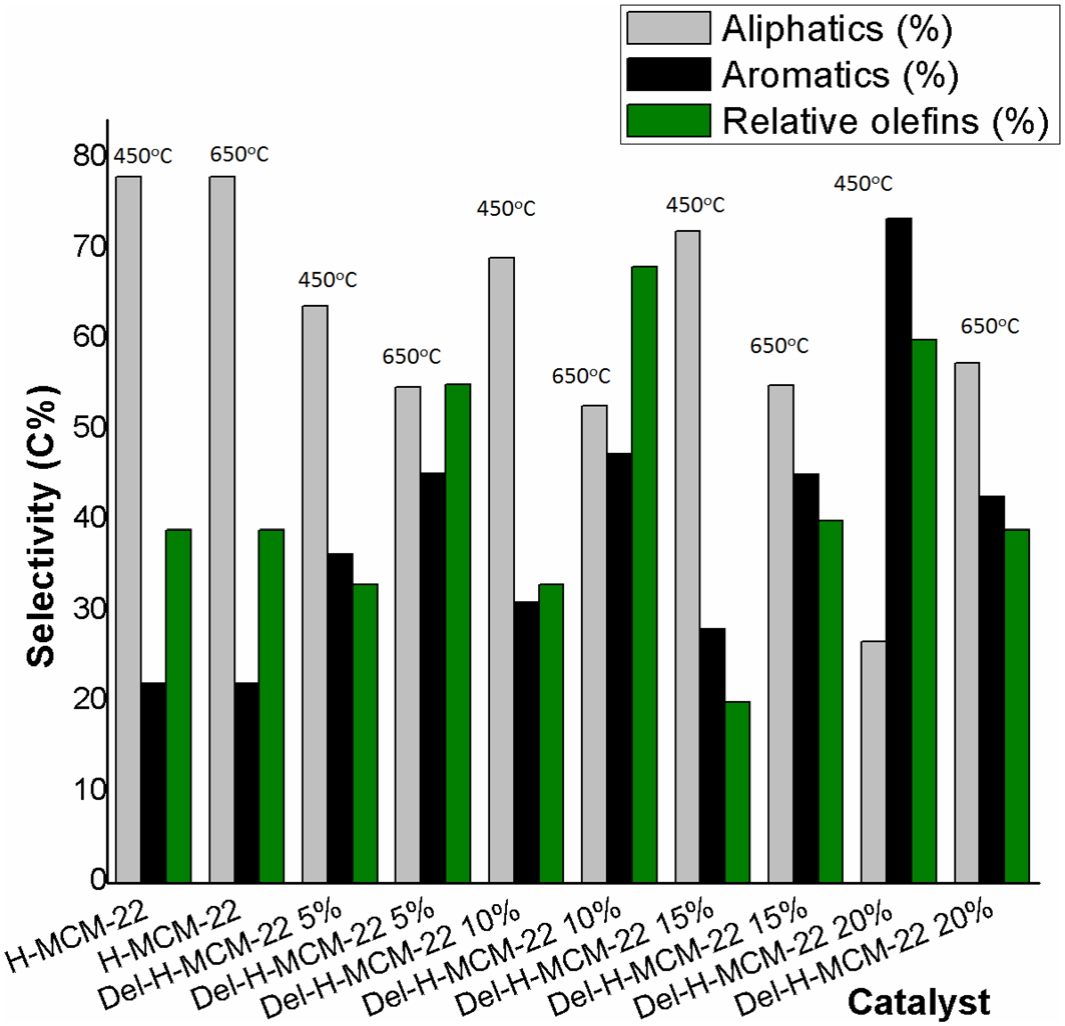
Comparison of aliphatic, aromatic and relative olefinic selectivity over prepared catalysts at 450 °C and 650 °C.

The n-Hexane transformation over H-MCM-22 zeolite at 450ºC and 650ºC is shown in Fig. 7. The distribution of products demonstrates the transformation of n-hexane into aliphatic compounds (78%) at temperatures 450ºC and 650ºC. However, the formation of olefin products is good at 650ºC as compared to 450ºC. The conversion of alkanes into olefins occurred at 650ºC. Further, the formation of light olefins is likewise extended with high reaction temperature. The higher reaction temperature complies with the most important monomolecular reaction mechanism because of which the conversion of higher alkanes (hexane, heptane) into lighter alkanes (propane) was also observed^13^.

The results of catalytic transformations of n-hexane over dealuminated H-MCM-22 zeolites are shown in Fig. 7. Results showed that dealumination favored the reduction of n-hexane transformation to aliphatic compounds at higher temperatures, which are consistent with the literature^13^. At 450ºC for H-MCM-22 5%, the alkanes with higher carbon numbers are produced but with less olefin production. The increase in the reaction temperature results in an increase in the selectivity of both higher and lighter olefins. The delamination of the catalyst caused the suppression of hydride transfer with an increase in reaction temperature, causing the cracking of heavier compounds into lighter products. In addition to this, it also increased the dehydrogenation reactions to produce low molecular weight compounds. The dealumination process is followed to lessen the number of acid sites due to which less active area is present for the reaction, in turn, causes the decrease of n-hexane conversion into aliphatic compounds and hence a decrease in coke formation^13^. As the concentration of oxalic acid is increased to 10%, the selectivity to lighter olefins increases with the decrease in conversion to aliphatic compounds. The comparison showed a reduction in aliphatic compounds from 69% to 52.69% from 450 °C to 650 °C. The reduction is because there is a suppression of hydride transfer due to the removal of acid sites from the structure of the zeolite. However, light olefins concentration was increased. In addition to this, H-MCM-22 10% displayed a comparative high BET surface area of 564 m^2^/g (Table 1) among the dealuminated structure. At higher cracking temperature i.e., 650 °C there is a removal of acid sites, which caused the formation of 3.4% 2-propanal of aliphatic compounds is due to the conversion of higher alkanes into olefins. Finally, the conversion to aliphatic compounds is reduced, but the selectivity is increased for olefins. With the increase in acid concentration, n-hexane conversion over H-MCM-22 15% reduced aliphatic compounds from 72% to 54.92%. The preliminary high conversion of H-MCM-22 15% could be due to strong acidic strength due to which less aluminum content is removed, which results in hydride transfer and an increase in paraffinic concentration. The selectivity for olefins increased with a rise in temperature at a high Si/Al ratio. H-MCM-22 20% showed the parallel behavior for the conversion of aliphatic compounds into olefins. Yet, the conversion to aliphatic compounds also increased with the rise in temperature, which can be related to the extent of delamination by using oxalic acid. As the concentration of oxalic acid increased, the dealumination causes the removal of extra framework aluminum atoms (EFAl)^8^. On the other hand, due to an increase in concentration, the nearby crystallites bind to surface hydroxyl groups causing the fragmentation of crystals.

#### ITQ-2

**Figure 8:**
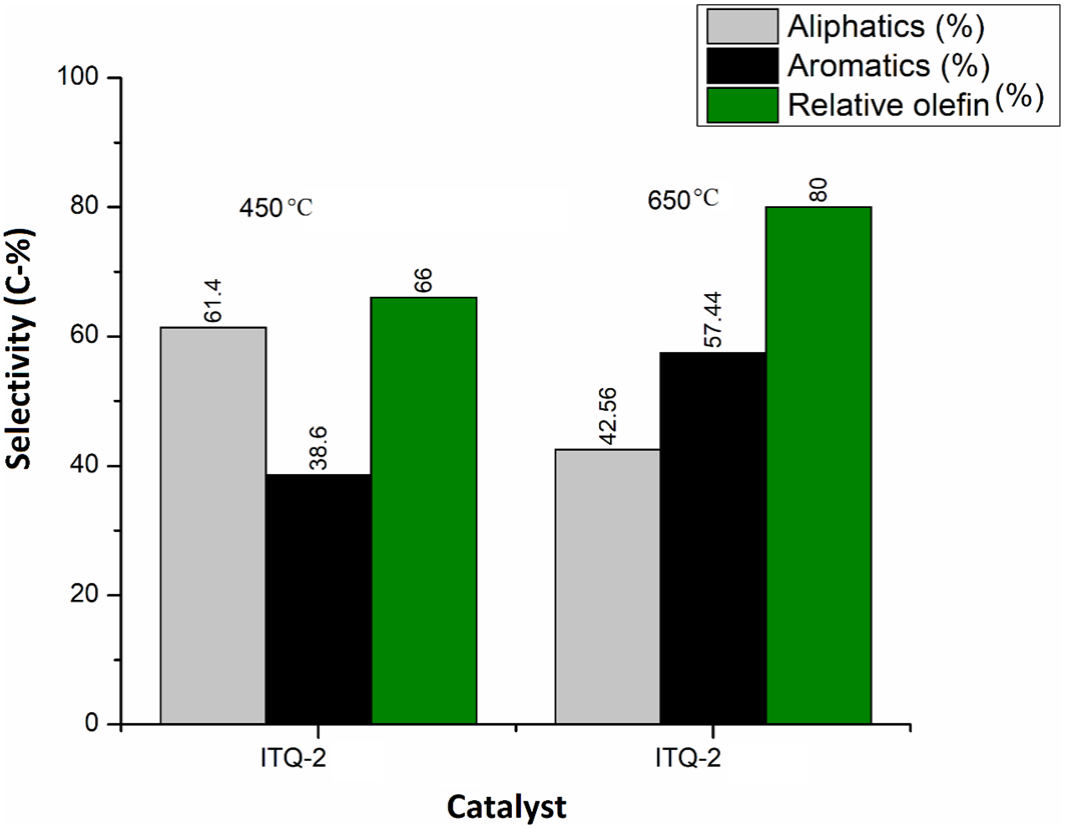
Comparison of aliphatic, aromatic and olefin selectivity over ITQ-2 zeolite at 450 °C and 650 °C.

The product distribution for ITQ-2 is shown in Fig. 8. The delaminated MCM-22 (ITQ-2) followed both cracking and reforming of n-hexane with a total aliphatic conversion of 61.4% at 450ºC. ITQ-2 followed the same behavior for n-hexane cracking as dealuminated structure; however, the purity % and quantity of light olefins was enhanced at a much higher rate. The rate of formation of propene, butenes, and several alkanes was enhanced at higher temperatures (650ºC), which is according to the literature^13^. A large number of aromatic compounds, including benzene, toluene, and xylene (BTX), were produced at both temperatures (450ºC and 650ºC) with the conversion of products from isomerization, reforming, and cracking reaction.

### Comparison of catalytic cracking of n-hexane over prepared zeolites

**Figure 9:**
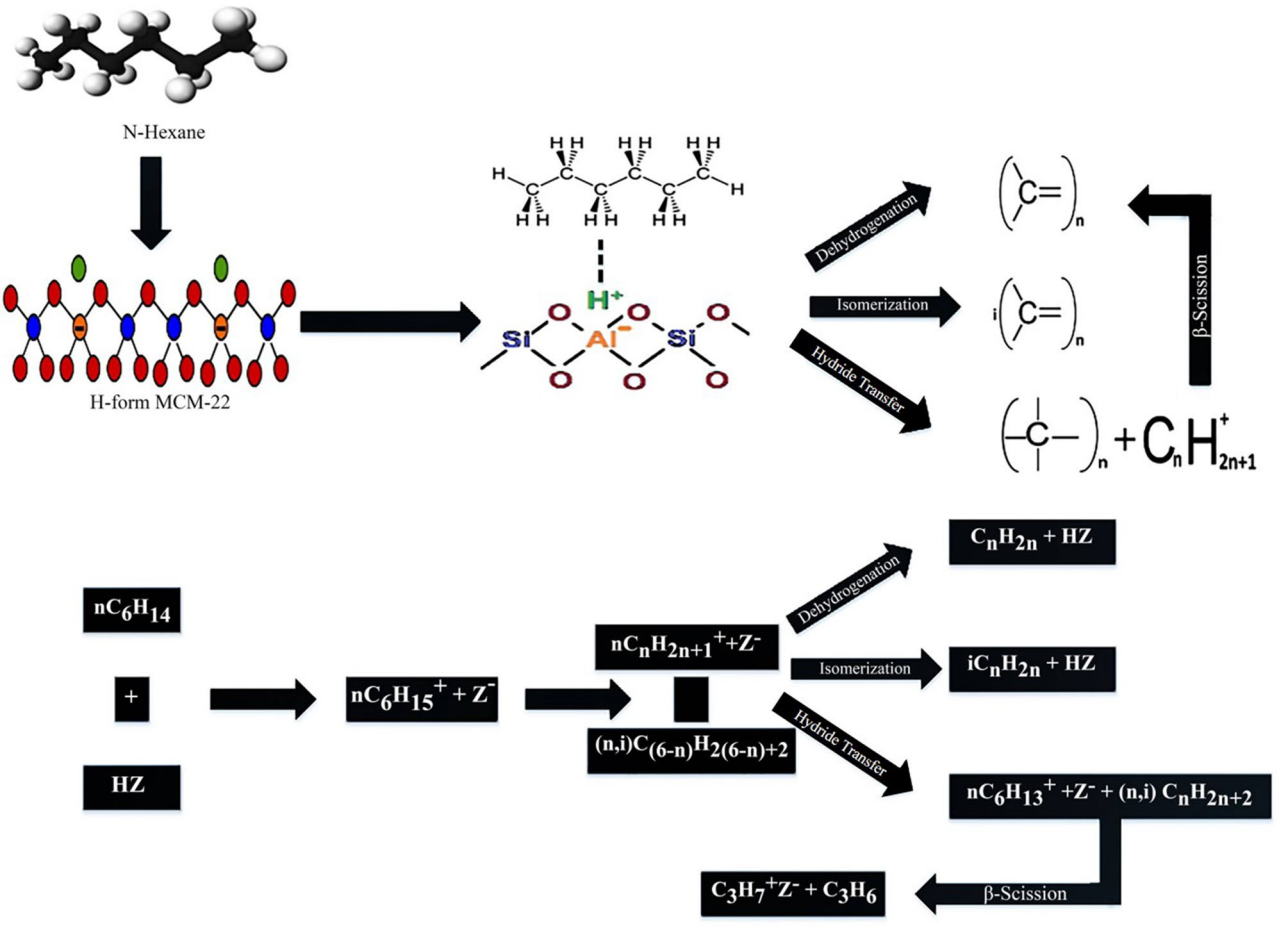
Reaction mechanism for n-hexane cracking over acidic zeolite.

Several studies are available for the catalytic cracking of alkanes over different zeolites as a model reaction of naphtha cracking. The performance of catalysts, reaction mechanisms, and structural effects are studied with low operating temperatures^31^. The catalytic cracking mechanism for paraffin follows the formation of a carbonium ion on the Bronsted acid site followed by decomposition in carbenium ion, which further undergoes dehydrogenation reaction and hydride transfer followed by β-scission to produce alkenes^32^. The reaction mechanism for catalytic cracking of n-hexane over acidic zeolite is shown in Fig. 9.

Table 2 shows the comparison of product distribution on different temperatures of 450 °C and 650 °C for the synthesized catalysts. It is observed that the number of aliphatic compounds was obtained (78%) using H-MCM-22 at both temperatures (450 °C, 650 °C). Among delaminated zeolites, the formation of more aromatics is accelerated by H-MCM-22 20% (73.33%) at 450ºC due to the binding of surface hydroxyl groups, which causes fragmentation of crystal surface. Due to this binding, surface basicity decreases, resulting in the re-adsorption of basic compounds, and the formation of aromatics increases.

**Table 2:** Product distribution over synthesized catalysts for n-hexane cracking.

S#	Catalyst	Temperature (°C)	Aliphatics (%)	Aromatics (%)	Rel.Olefins (%)
1	MCM-22	450	61.6	38.40	33
		650	62.07	37.93	43
2	H-MCM-22	450	78	22	39
		650	78	22	39
3	H-MCM-22 5%	450	63.67	36.33	33
		650	54.78	45.22	55
4	H-MCM-22 10%	450	69	31	33
		650	52.69	47.31	68
5	H-MCM-22 15%	450	72	28	20
		650	54.92	45.08	40
6	H-MCM-22 20%	450	26.67	73.33	60
		650	57.37	42.63	39
7	ITQ-2	450	61.40	38.6	66
		650	42.56	57.44	80

For olefins production, the delaminated MCM-22 (ITQ-2) structure gives the highest conversion of up to 80% relative olefins at 650 °C. The transformation to light olefins increases with the rise in temperature, and several light olefins are produced. Furthermore, ITQ-2 has fewer acid sites than MCM-22 and its dealuminated zeolites. Therefore, less hydride transfer is accelerated, and the secondary reactions are controlled. Among the dealuminated structure, the highest chance to relative olefins is obtained over H-MCM-22 10% at 650ºC. The conversion of paraffinic concentrations to olefins is accounted for by control over acid sites by removing the Al atoms, which is the reason for the acidity in zeolites. By removing Al atoms, the acid amount decreases and causes less hydride transfer and secondary reactions, which increases olefins concentration by controlling bimolecular reactions. The highest transformation to relative olefins over H-MCM-22 10% was 68%.

## Conclusion

MCM-22 catalyst, along with its derivative structures, showed comparable results for n-hexane cracking. Almost every catalyst showed higher selectivity in transformation of alkanes into olefins at higher temperatures. H-MCM-22 10% provided the highest olefins concentration (68%) among de-aluminated H-MCM-22 zeolites owing to the efficient removal of acid amounts that suppress the hydride transfer for secondary reactions. H-MCM-22 15% and H-MCM-22 20% showed a decrease in relative olefins concentration due to a rise in oxalic acid concentration that causes the binding of nearby crystallites. Moreover, ITQ-2 showed higher olefins selectivity and produced light olefins at higher temperatures with relative olefins concentration of 80% as a result of the specific short pore structure of ITQ-2 that restricts the formation of heavy aliphatic compounds and increases the selectivity for light olefins.
