# Author Correction: Synthesis, characterization and catalytic testing of MCM-22 derived catalysts for n-hexane cracking

**DOI:** 10.1038/s41598-021-88093-y

**Published:** 2021-04-22

**Authors:** Ali Ahmad, Salman Raza Naqvi, Muhammad Rafque, Habib Nasir, Ali Sarosh

**Affiliations:** 1 School of Chemical and Materials Engineering, National University of Sciences and Technology, H-12, Islamabad, 44000 Pakistan; 2 Department of Chemical Engineering, University of Gujrat, Hafz Hayat Campus, Gujrat, Pakistan; 3 Quaid- I-Azam University, Islamabad, Pakistan; 4 Department of Chemistry, School of Natural Sciences, National University of Sciences and Technology, H-12, Islamabad, 44000 Pakistan; 5 Muhammad Nawaz Sharif University of Engineering and Technology (MNS-UET), Multan, Pakistan

Correction to: *Scientific Reports* 10.1038/s41598-020-78746-9, published online 11 December 2020

The original version of this Article omitted an affiliation for Ali Ahmad. The correct affiliations for Ali Ahmad are listed below:

School of Chemical and Materials Engineering, National University of Sciences and Technology, H-12, Islamabad, 44000, Pakistan

Department of Chemical Engineering, University of Gujrat, Hafiz Hayat Campus, Gujrat, Pakistan

